# Novel Therapies for Relapsed or Refractory Diffuse Large B-Cell Lymphoma

**DOI:** 10.3390/ijms21228553

**Published:** 2020-11-13

**Authors:** Leonard Jeff Harris, Kruti Patel, Michael Martin

**Affiliations:** 1 Oncology Division, Department of Medicine, University of Tennessee Health Sciences Center, Memphis, TN 38103, USA; kpatel@WESTCLINIC.com; 2 West Cancer Center & Research Institute, Memphis, TN 38103, USA; mmartin@WESTCLINIC.com

**Keywords:** Relapsed or Refractory Diffuse Large B Cell Lymphoma, DLBLC, immunotherapy, chemotherapy-free regimen

## Abstract

The most common type of non-Hodgkin lymphoma in adults is diffuse large B-cell (DLBCL). There is a historical unmet need for more effective therapies in the 2nd and 3rd line setting. Emerging immunochemotherapies have shown activity in small studies of heavily pre-treated patients with prolonged remissions achieved in some patients. Anti-CD19 CAR (chimeric antigen receptor) T cells are potentially curative in the 3rd line and beyond setting and are under investigation in earlier lines of therapy. Antibody-drug conjugates (ADC’s) such as polatuzumab vedotin targeting the pan-B-cell marker CD79b has proven effectiveness in multiply-relapsed DLBCL patients. Tafasitamab (MOR208) is an anti-CD19 monoclonal antibody producing prolonged remissions when combined with Lenalidomide (LEN) in patients who were not candidates for salvage chemotherapy or autologous stem cell transplant. Selinexor, an oral, small-molecule selective inhibitor of XPO1-mediated nuclear export (SINE), demonstrated prolonged activity against heavily-pretreated DLBCL without cumulative toxicity and is being investigated as part of an oral, chemotherapy-free regimen for relapsed aggressive lymphoma. This article reviews current strategies and novel therapies for relapsed/refractory DLBCL.

## 1. Introduction

Diffuse large B-Cell Lymphoma (DLBCL) is an aggressive subtype accounting for 25–30% of Non-Hodgkin lymphoma (NHL) with an incidence of 5.6 per 100,000 persons per year [1,2]. DLBCL is usually symptomatic at presentation with either nodal or extranodal disease. Diagnosis is made when large, transformed B cells (CD19+, CD20+, CD79+) with prominent nucleoli, diffuse growth pattern, and a high proliferation fraction are seen on tissue biopsy [1]. The World Health Organization (WHO) schema classifies by cell of origin (COO) classification including germinal B-cell (GCB) subtype or activated B cell (ABC) subtype, but more recent transcriptome sequencing techniques have identified five distinct subtypes that improve the differentiation among prognostic groups in DLBCL [3,4]. Genomic instability is demonstrated by a median of 17 (range: 0–48) genetic drivers that were clustered into these 5 distinct genetic signatures. In the 2017 revision of WHO classifications, DLBCL with translocations of MYC and BCL2 and/or BCL6—double-hit (DHL) or triple-hit (THL)—are reclassified as Diffuse Aggressive B-Cell Lymphomas, with more intense therapeutic regimens such as DA-EPOCH-R with CNS prophylaxis in the first line setting having a 4 year overall survival of 72.2% [5]. Despite multiple studies attempting to improve upon the outcomes, R-CHOP remains the first line treatment for DLBCL regardless of IPI score, COO, or gene expression profile except for DHL.

DLBCL cases that do not fit a specific subtype have an overall survival rate of 65% when treated with standard R-CHOP (Rituximab, Cyclosporine, Vincristine, Prednisone) therapy [1]. The Standard International Prognostic Index (IPI) is widely used for risk stratification with aggressive B-cell lymphoma, and has been validated with continued prediction of risk in the Rituximab era [6]. Patients with a high IPI score have poor prognosis with an OS as low as 20–25%. Certain mutations and pathways are common in the GCB subtype such as EZH2, BCL2 and PI3K. In the ABC subtype, NF-KB activation, MYD88 mutations and JAK-STAT pathways are more common [7].

While most patients respond, 30–40% of patients with DLBCL relapse or are unable to achieve remission with first-line treatment. In these cases, the prognosis is poor [8]. Approximately 50% of patients with relapsed or refractory (R/R) DLBCL have a response to second-line chemotherapy; up to 50% of these patients proceed to undergo autologous hematopoietic stem-cell transplantation in some settings, and of these, approximately 30 to 40% remain progression-free 3 years after transplantation [8,9,10,11,12]. Median survival for primary and secondary refractory DLBCL is 5–7 months [8].

Patients who progress after receiving R-CHOP receive combination salvage chemotherapy. Commonly used regimens, including R-ICE, R-DHAP, R-GDP, R-GemOx, O-DHAP, O-ICE, and DR-ICE, have similar treatment effects [13,14]. However, analysis of real-world data from 126 community-based hematology/oncology practices in the US between 2010–2016 demonstrated that only 13% of patients who received salvage regimens intended for ASCT eventually underwent ASCT [15].

The unmet need for more effective regimens is highlighted by the wide heterogeneity in regimens used in clinical practice with consistently poor outcomes [13]. Pts that are not candidates for ASCT and those who never make it or have failed CAR-T therapy have poor outcomes with salvage chemotherapy regimens. Response rate comparisons between studies are unreliable due different rates of enrollment of primary refractory disease. In the phase III CORAL trial (*n* = 396) comparing R-ICE and R-DHAP followed by autologous hematopoietic cell transplant (HCT) for chemosensitive patients, the overall response was 63%, and the three year overall survival was 47%. Median overall survival of R/R DLBCL who failed second-line regimens in CORAL was 4.4 months [8]. The LY.12 trial (*n* = 619) compared the platinum-containing regimens R-GDP and R-DHAP followed by autologous HCT and had response rate 45% [12]. SCHOLAR-1 is the most comprehensive analysis of pooled outcomes from several large studies of relapsed and refractory DLBCL (*n* = 636) treated with various standard of care chemotherapy regimens, and the ORR was 26%, CR rate of 7%, and median overall survival was 6.2 months [16,17].

A cost-effectiveness analysis of DLBCL regimens from the Truven database with claims data from US government and private payers highlighted the direct costs associated with the 2/3 of patients with DLBCL who received subsequent 2nd-line regimen after completing R-CHOP [18]. More effective treatment options for this resource intensive condition has the potential to both decrease mortality and reduce the costs of subsequent lines of therapy including ASCT [18,19]. Several innovative treatment modalities are already receiving regulatory approvals (Table 1).

## 2. Tafasitamab

Tafasitamab (MOR208) is an Fc-enhanced, humanized, anti-CD19 monoclonal antibody that has shown preclinical and single-agent activity in patients with relapsed or refractory B-cell malignancies [23]. It has been engineered to have better antibody directed cellular cytotoxicity (ADCC) than a native antibody LEN enhances natural killer cell-meditated, antibody-dependent cellular cytotoxicity with tafasitamab in vitro [24]. A recently published analysis confirmed synergistic effects of combining tafasitamab with LEN by comparing the L-MIND study (combination) to RE-MIND (LEN monotherapy) [25]. L-MIND is an open-label, single-arm, phase II clinical trial of tafasitamab plus lenalidomide in patients with relapsed or refractory DLBCL who were ineligible for high-dose chemotherapy with autologous stem-cell transplantation due to factors such as advanced age, refusal, or comorbidities [22]. Double-hit (simultaneous detection of *MYC* with *BCL2* or *BCL6* translocation) and primary refractory patients who relapsed within 6 months of anti-CD20 therapy were excluded (in the first 6 months of recruitment the exclusion was only 3 months to be primary refractory). Of the 80 patients who received the dual therapy, 43% experienced complete responses and 18% exhibited partial responses, and the median duration of these responses was 21.7 months, which is in contrast to LEN monotherapy in similar patients, where only 13.2% experienced complete response [25], and in contrast to the R2 regimen of LEN and Rituximab for R/R DLBCL, where only 13.3% experienced complete response [26]. All Tafasitamab+LEN patients experienced treatment-emergent adverse events, with neutropenia being the most common adverse event. Nonhematologic adverse events were most often grade 1 or 2 and included diarrhea and rash [22]. Notably, 12% of patients discontinued study treatment because of adverse events, and four patients died of treatment-emergent adverse events, although none of these deaths were deemed by the investigators to be due to the study treatment [27]. However, cytopenias are likely related to LEN use. Now in a phase 1b study, MOR 208 is being studied in combination with R-CHOP and lenalidomide for frontline DLBCL treatment.

## 3. Polatuzumab Vedotin

Polatuzumab vedotin is an antibody–drug conjugate (ADC) consisting of a humanized anti-CD79b monoclonal antibody and the anti-mitotic agent, mono-methyl auristatin E (MMAE) [28]. This Polatuzumab vedotin’s antibody recognizes the CD79b protein that is associated with the B-cell receptor. After the antibody binds to CD79b, the ADC’s toxic payload (MMAE) enters the B cell and then kills it by preventing tubulin polymerization. Targeting the pan-B marker CD79b is ideal since it will not select for resistance to CD19 regimens for patients who may later require CAR T cell therapy directed against CD19 [29]. Given as a parenteral triplet, polatuzumab-vedotin plus bendamustine and rituximab is approved for third-line therapy use after demonstrating a median overall survival of 12.4 months compared with 4.7 months for patients receiving a current standard salvage regimen of bendamustine and ritixumab [20]. Patients were excluded if they had a history of grade ≥2 peripheral neuropathy or prior HSCT. Primary-refractory nor double/triple-hit lymphomas were excluded. Adverse reactions led to dose reduction in 18%, dose interruption in 51%, and permanent discontinuation of all treatment in 31%. The most common adverse reactions leading to treatment discontinuation were thrombocytopenia and/or neutropenia [30]. In early phase studies and clinical practice, experts suggest Polatuzumab has significant single agent activity and it can be given without bendamustine. The POLARGO study is a currently enrolling a multicenter phase III randomized controlled trial of pola-R-GemOx vs. R-GemOx alone in R/R DLBCL [31]. In patients with relapsed disease who need a bridge to either CAR-T or ASCT, polatuzumab has proven to be an active regimen used in this situation. Since polatuzumab has been effective and well tolerated, there is a currently ongoing trial, POLARIX, using it in upfront therapy with RCHOP.

## 4. Selinexor

Selinexor, an oral selective inhibitor of XPO1-mediated nuclear export (SINE), has a broad potential mechanism of action. It induces the expected nuclear accumulation and activation of tumor suppressor proteins and reduces Bcl2, Bcl-X_L_, and c-Myc oncoprotein concentrations [21]. It received accelerated FDA approval in 2019 for relapsed or refractory DLBCL after 2 lines of systemic therapy in addition to approval for relapsed Multiple Myeloma [32]. In the multicenter, open-label, phase 2b SADAL study, 127 patients with DLBCL who had received two to five lines of previous therapies, and progressed after or were not candidates for autologous stem-cell transplantation were given selinexor orally at the fixed dose of 60 mg on day 1 and day 3 weekly, until disease progression or manifested unacceptable toxicity [33]. The primary endpoint of overall response rate was 28% (36/127) with median duration of response 23 months. Complete response was achieved in 12% with a median duration of 23.0 months. In the subgroup analysis of those with low c-myc expression by immunohistochemistry, the overall response rate was 42% [21]. Selinexor caused adverse events that were reversible with standard supportive care and dose modification to 40 mg dosing. Without apparent cumulative toxicity, there is currently no maximum duration of treatment; the longest duration of treatment in the SADAL study with selinexor is > 3.5 years [21]. Two experimental studies (NCT02303392 and NCT03955783) in aggressive lymphoma, testing the combinations of selinexor with ibrutinib or venetoclax are active and recruiting. Results of the studies might clarify if a totally oral and chemotherapy free treatment should be an option for patients with relapsed or refractory DLBCL.

## 5. CAR T Cells

Anti-CD19 CAR T cell therapy has transformed the approach to multiple-relapsed/refractory aggressive B-cell lymphomas. Three anti-CD19 CAR T cell products have demonstrated efficacy in relapsed DLBCL with a remarkably long duration of effect in patients who achieve a complete response. Second generation receptors, dual target CD19/CD22, novel dose escalation protocols, and addition of PD-1 blockade are in ongoing studies for improved efficacy and/or reduced toxicity [34]. The indications for use may expand in the future as the collection and manufacturing process becomes more streamlined and more centers develop experience managing its toxicities [9,35,36,37]. Currently, axicabtagene ciloleucel (axi-cel) and Tisagenlecleucel (tisa-cel) are approved by the US Food and Drug Administration in adults with relapsed or refractory DLBCL after two or more lines of systemic therapy. The ZUMA study (*n* = 101) of axi-cel reported a 58% CR and 24 month survival rate of 50.5%. The cohort of patients age ≥ 65 years (*n* = 81) had a 44% rate of grade ≥3 neurologic toxicity. The JULIET study (*n* = 93) of tisagenlecleucel had a 40% CR and 12 month overall survival rate of 49%. The TRANSCEND study (*n* = 344) of lisocabtagene maraleucel reported CR 53% and estimated 12 month overall survival rate 58%, and only 1% experienced grade 3 or higher neurologic toxicity of confusional state during therapy [38]. Multiple randomized trials are currently enrolling patients with primary refractory or early relapsed aggressive B-cell lymphomas comparing anti-CD19 CAR T cell therapy with traditional salvage therapy and ASCT (TRANSFORM, NCT03575351; BELINDA, NCT03570892; and ZUMA-7, NCT03391466) [39]. A key limitation of CAR-T therapies is limitations in access and the time that lapses between collecting and infusing cells that ranged 17 to 54 days during phase 2 clinical trials [40].

## 6. Sequencing Therapy

With recent new drug approvals, treatment options for patients with R/R DLBCL have expanded. However, this poses a challenge in sequencing and treatment selection for patients. At this time, the sequencing of therapy is individualized based on the efficacy and side effect profile of treatment. In patients with R/R DLBCL, the treatment should be divided among transplant eligible and ineligible patients. If they are transplant ineligible or progress after ASCT, they have all the above approved regimens available as option. ASCT ineligible patients should be evaluated for CAR-T therapy as it offers the best ORR among therapies mentioned in Table 1. However, CAR-T can be challenging in terms of accessibility, the patient’s functional status, disease burden and other factors. Polatuxumab Vedotin in combination with bendamustine and rituximab is another option and can be used as a bridge to CAR-T as well. Polatuzumab Veodtin in combination with BR had 40% of CRR and manageable toxicities [20]. If patients respond well, Bendamustine can be dropped to allow cell collection for CAR-T. However, it is a three drug regimen and it carries risk of grade 3 or 4 cytopenias and peripheral neuropathy. In patients that are not candidates for CAR-T and goal is palliation, Tafasitamab with lenalidomide is a great option with limited toxicities. Based on the L-Mind study, Tafasitamab + Len had ORR of 60% and CRR of 42.5% and main side effects were cytopenias managed by dose adjustment of lenalidomide [22]. Tafasitamab prior to CAR-T may alter efficacy of CAR-T therapy since they both are CD-19 targeted therapy; however, more data are needed to support this. Selinexor is another option for ASCT ineligible patients with ORR of 28%; relatively lower than other agents. Selinexor also has a significant side effect profile for GI toxicity, hyponatremia and cytopenia, hence would reserve this as a last option.

## 7. Future of DLBCL and Immunotherapy

There are many other immunotherapy based regimens under early clinical trials aside from those mentioned above (Table 2).

MT-3724 is a novel Engineered Toxic Body (ETB) comprised of a proprietarily engineered form of Shiga-like Toxin A subunit (SLT-A) genetically fused to an antibody-like binding domain that binds CD20. ETBs work though a novel mechanism of action whereby the internalization of the fragment when bound to CD20 delivers the toxin intracellularly where ribosomal inactivation leads to targeted cell death [50,51]. MT-3724 is currently being studied in three ongoing Phase 2 studies for relapsed and refractory DLBCL. Loncastuximab tesirine, ADCT-402 is an antibody-drug conjugate composed of a humanized monoclonal antibody against CD19 and conjugated to a pyrrolobenzodiazepine dimer cytotoxin. In phase 2 trials, ADCT-402, 145 pts with relapsed or refractory DLBCL were enrolled and ORR was 45%. The common side effects were cytopenias requiring dose adjustments, which were otherwise well tolerated. Hu5F9-G4, a humanized monoclonal antibody is a macrophage immune checkpoint inhibitor blocking CD47 that induces tumor-cell phagocytosis. A phase 1B study, 22 pts with relapsed NHL were treated with Hu5F9-G4 in combination with rituximab. The ORR in DLBCL subset was 40% with CR of 33%. The most common AEs were infusion reaction, fever and chills. Immune checkpoint inhibitors have gained recognition in multiple solid tumors and demonstrated durable responses. PD-1 and PDL-1 are expressed in many hematologic malignancies and have recently been approved for second line HL. In a phase 1 trial of relapsed DLBCL patients, nivolumab showed an ORR of 36%, but these responses were not durable. There are a few trials in DLBCL being completed with immune checkpoint inhibitors in combination with anti-CD-20 antibodies (NCT03401853) and immunomodulators and targeted agents such as LEN (NCT03015896) and Copanlisib (NCT03484819). Table 1 includes a list early clinical trials involving immunotherapy for treatment of relapsed/refractory DLBCL.

## 8. Conclusions

Novel agents are changing treatment strategies in relapsed or refractory DLBCL after the failure of cytotoxic chemoimmunotherapy. Harnessing the surveillance of the patient’s T cell immunity has produced prolonged responses in studies of heavily pre-treated patients. The inclusion of immunotherapies such as CAR T cells, bispecific antibodies, ADCs, and other immunomodulatory drugs to the treatment algorithms for DLBCL is filling the unmet need for agents with activity in the multiply relapsed setting. A recurring theme in the development of noncytotoxic regimens is that chemotherapy free does not equal toxicity-free [39]. Distinct adverse effects seen with immunotherapies are part of these treatment decisions, and ongoing studies are informing the physical toxicities expected in broader populations. Learning which patients reap the most benefit from these agents enables more accurate calculations of financial toxicities.

## Figures and Tables

**Table 1:** Novel Regimens with FDA Approval.

Agent	Year of FDA Approval	Regimen	Population	Relapse < 1 year of DLBCL Diagnosis	Refractory to Last Regimen	DHL/THL	Efficacy Outcomes
Axicabtagene ciloleucel (axi-cel)	2017	Flu/Cy LD	R/R DLBCL refractory to 2 lines of therapy	30%	77%	NR	ORR 83% CR 58% mOS 24 mos
Lisocabtagene maraleucel		Flu/Cy LD	R/R DLBCL refractory to 2 lines of therapy	NR	44%	13%	ORR 73% CR 53% mOS >12 mos
Tisagenlecleucel ^a^	2018	Flu/Cy LD or Benda-Flu LD	R/R DLBCL refractory to 2 lines of therapy	NR	40%	27%	ORR 52 CR 40% mOS 12 mos
Polatuzumab vedotin [20]	2019	Pola + BR	R/R DLBCL Ineligible for ASCT	53%	75%	0%	CMR 40% mOS 12.4 mos
Selinexor [21]	2020	Selinexor 60 mg po on days 1 and 3 of each week	R/R DLBCL	33% §§	72%	4%	ORR 28% CR 12% mOS 9.1 mos
Tafasitamab [22]	2020	Tafa + LEN 25 mg	R/R DLBCL Ineligible for ASCT	19% §	44%	0%	ORR 58% CR 33% mOS 22 mos

FDA: United States Food and Drug Administration; Flu/Cy: Fludarabine/Cyclophosphamide; LD: lymphodepletion; Benda/Flu: Bendamustin/Cyclophosphamide; Pola: Polatuzumab vedotin; BR: Bendamustin and Rituximab; Ritux: Rituximab; LEN: Lenalidomide; Tafa: Tafasitamab; dx: diagnosis; DHL: Double Hit Lymphoma; THL: Triple Hit Lymphoma; R/R DLBCL: Relapsed or Refractory Diffuse Large B Cell Lymphoma; ORR: Overall Response Rate; CR: Complete Response; mOS: Median Overall Survival; mos: months; CMR: Complete Metabolic Response; po: by mouth; NR: Not Reported. ^a^: investigational agent with pending Food and Drug Administration approval. § Excluded if received anti-CD20 therapy within 6 months. §§ Excluded if not in PR or CR and received therapy within 14 weeks.

**Table 2:** Novel Regimens under Investigation for Relapsed or Refractory Diffuse Large B Cell Lymphoma.

**Bispecific Abs**				
Epcoritamab (CD3/CD20) Flat dose Subcutaneous weekly Escalation study	Hutchings et al. [41] NCT03625037	Phase 1/2 R/R DLBCL	*N* = 41	Enrolling Median f/u 4.7 mo ORR 56% CR 44% No dose limiting toxicities
Odronextamab REGN1979 (CD3/CD20) 18–320 mg doses	Bannerji et al. [42] NCT03888105	Phase 1 R/R DLBCL	*N* = 19	Enrolling phase 2 ORR 58% CR 37%
Monsenetuzumab (CD3/CD20)	Schuster et al. [43] NCT03677154	Phase 1/2 R/R DLBCL including p CAR-T	*N* = 119	Enrolling phase 3 ORR 34.7% CR 18.6%
Glofitamab RG6026 (CD3/CD20)	Morschhauser et al. [44] NCT03075696	Phase 1/Ib R/R aggressive NHL +/− Obinituzumab	*N* = 21	Enrolling Phase 1 ORR 38% CR 31%
**Monoclonal Abs**				
Tafasitamab (anti-CD19) (Fc-enhanced, humanized) +Lenolidomide	Nowakowski et al. [25] Maddocks et al. [45] NCT02399085	Phase 1/2 R/R DLBCL Ineligible for ASCT Excluded double-hit	*N* = 81	Enrolling phase 3 ORR 58% CR 33% Median OS 22 mos (95% CI: 18.6–NR)
Magrolimab (5F9) (anti-CD47, promote phagocytosis) +Rituximab	Advani et al. [46] NCT02953509	Phase 1b/2 R/R DLBCL	*N* = 15	Enrolling, Preliminary results ORR 40% CR 27% On-target anemia primarily 1st dose
**Anti-PD-L1 Containing Regimens**				
Atezolizumab (anti-PDL1) +Obinituzumab (anti-CD20) +Venetoclax (BCL2 inhibitor)	Herbaux et al. [47] NCT03276468	Phase 2 R/R DLBCL	*N* = 58	Interim Results ORR 23.6% CMR 18%
Mogamulizumab (anti-CCR4) +Pembrolizumab	Joffe et al. NCT03309878	Phase 1b/2 R/R DLBCL Ineligible for ASCT		Enrolling
Avelumab (anti-PD-L1) +/− Utomilumab (4-1BB agonist) +/− Rituximab +/− Bendamustine or Azacitidine	Chen et al. [48] NCT02951156	Phase 1b/3 R/R DLBCL Ineligible for ASCT ECOG ≤ 1		Enrolling
**Bispecific CAR T Cell Therapies**				
AUTO3 (CD19/CD22) Dual targeted +Pembrolizumab	Osborne et al. NCT03287817	Phase 1/2 R/R DLBCL	*N* = 11	ORR 64%CRR 55%
LV20.19CAR (CD19/CD20) Dual targeted Lentiviral	Shah et al. [49] NCT03019055	Phase 1 R/R NHL 45% DLBCL		Enrolling in expansion phase ORR 82% CR 54.5% No grade 3–4 CRS or NTX in first 11 pts.
**Antibody-Drug Conjugates**				
Polatuzumab vedotin (anti-CD79b/MMAE) added to BR	Sehn et al. [20] Lu et al. [28] NCT02257567	Phase 2 R/R DLBCL Ineligible for ASCT	*N* = 80	CMR 40% Median OS 12.4 mos
Polatuzumab vedotin (anti-CD79b/MMAE) added to Gem-Ox	Haioun et al. [31] NCT04182204	Phase 3 R/R DLBCL		Enrolling
**Engineered Toxin Bodies**				
MT-3724 (CD20/ SLT-I A1)	Fanale et al. [50] Duque et al. [51] NCT02361346	Phase 1 Relapsed B-NHL after anti-CD20 and CT	*N* = 100	Safety and efficacy assessment of 50 mcg/kg/dose ongoing.
**PI3K Inhibitor**				
Parsaclisib 20 mg po daily	Coleman et al. [52,53] NCT02998476	Phase 2 R/R DLBCL	*N* = 60	Interim Results ORR 25% CMR 12.5%
Buparlisib 80 mg po daily +Ibrutinib	Batlevi et al. [54] NCT02756247	Phase 1/2 R/R DLBCL, Mantle Cell, Follicular	*N* = 37	Interim Results ORR 31% CMR 23%
**BTK Inhibitors**				
Acalabrutinib 100 mg po BID +Pembrolizumab	Witzig et al. [55] NCT02362035	Phase 1/2 R/R DLBCL	*N* = 61	ORR 26% CR 7%
Zanubrutinib 160 mg po BID	Yang et al. [56] NCT03145064	Phase 2 R/R Non-GBC DLBCL Ineligible for ASCT	*N* = 41	ORR 29.3% CR 17.1% Median OS 8.4 mos
**Immunomodulators**				
R2-GDP Lenalidomide 10 mg po d1-14 + R-GDP	Merino et al. [57] EudraCT 2014-001620-29	Phase 2 R/R DLBCL Ineligible for ASCT	*N* = 79	Enrolling ORR 59% CR 32% Median OS 12 mos
R2-ICE Lenalidomide 20 mg po d1-14 + RICE	Guerra-Bauman et al. [58] NCT02628405	Phase 1/2 R/R DLBCL Candidates for ASCT		Enrolling

PO = by mouth; BID = twice daily; Mo(s) = month(s); ORR = Overall Response Rate; CR = Complete Response; CMR = Complete Metabolic Response by Positron Emission Testing (PET); CT = Chemotherapy; BR = Bendamustin/Rituximab; Gem-Ox = Gemcitabine/Oxaliplatin; AE Trmt DC Ac/Pem = Adverse Events causing Treatment Discontinuation due to Acalabrutinib/Pembrolizumab; SLT-I A1 = Shiga-like toxin-I A1; R2-GDP (Lenalidomide, Rituximab, Gemcitabine, Dexamethasone, Cisplatin); R2-ICE (Lenalidomide, Rituximab, Ifosfamide, Carboplatin, Etoposide).
